# Supplementary material for: Lactide and Ethylene Brassylate-Based Thermoplastic Elastomers and Their Nanocomposites with Carbon Nanotubes: Synthesis, Mechanical Properties and Interaction with Astrocytes
Source: Polymers (Basel). 2022 Nov 1;14(21):4656. doi: 10.3390/polym14214656 (PMC9658163; doi:10.3390/polym14214656)
Supplement: Supplementary file 1 [file polymers-14-04656-s001.zip › polymers-1998623-supplementary.pdf]

polymers

MDPI

Supplementary Materials for:

Lactide and Ethylene Brassylate-Based Thermoplastic Elastomers and Their Nanocomposites with Carbon Nanotubes: Synthesis, Mechanical Properties and Interaction with Astrocytes

Carlos Bello-Álvarez [1], Agustin Etxeberria [2], Yurena Polo [3], Jose-Ramon Sarasua [1], Ester Zuza [1,*] and Aitor Larrañaga [1,*]

[1] Department of Mining-Metallurgy Engineering and Materials Science, POLYMAT, Faculty of Engineering in Bilbao, University of the Basque Country (UPV/EHU), Plaza Ingeniero Torres Quevedo 1, 48013 Bilbao, Spain
[2] Advanced Polymers and Materials: Physics, Chemistry and Technology Department, POLYMAT, University of the Basque Country (UPV/EHU), 20018 Donostia-San Sebastián, Spain
[3] Polimerbio SL, 20014 Donostia-San Sebastian, Spain
* Correspondence: ester.zuza@ehu.eus (E.Z.); aitor.larranagae@ehu.eus (A.L.); Tel.: +34-946013925 (E.Z.); +34-946013935 (A.L.)

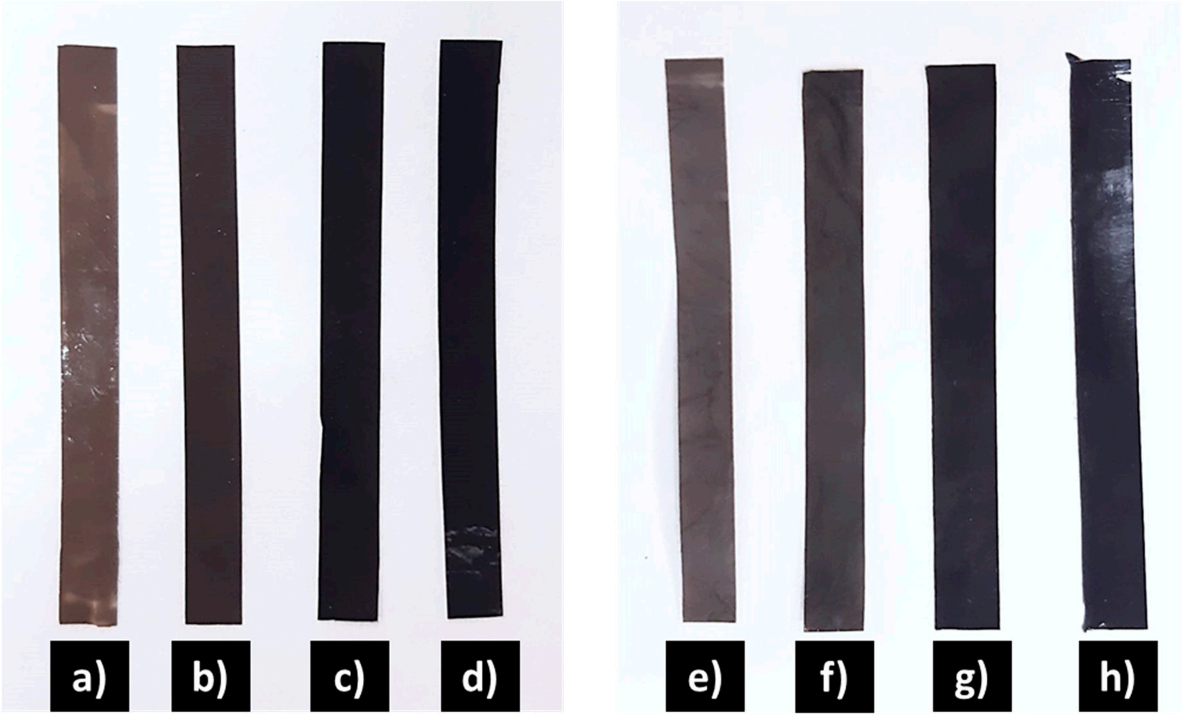

Figure S1. DL:EB samples for tensile testing with different amounts of CNT: a) 0%CNT; b) 0.1%CNT; c) 0.5%CNT and d) 1%CNT. L:EB samples for tensile testing with different amounts of CNT: e) 0%CNT; f) 0.1%CNT; g) 0.5%CNT and h) 1%CNT.

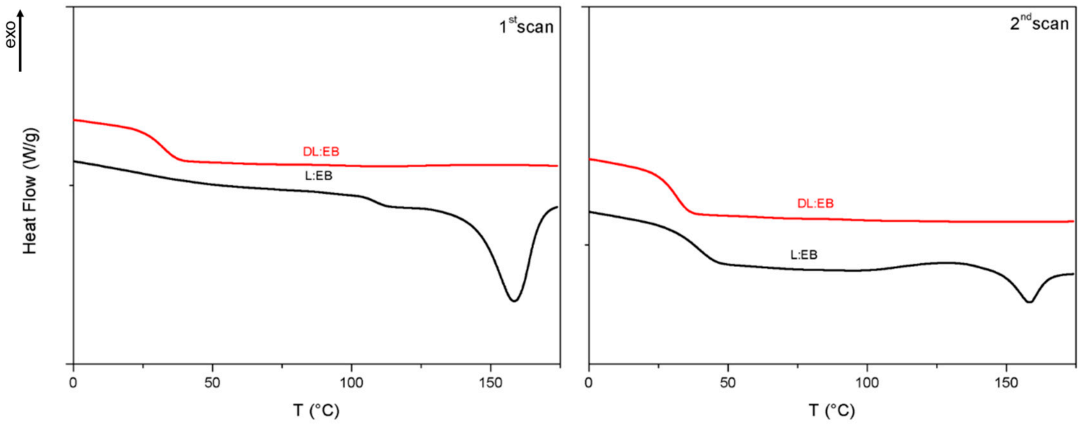

Figure S2. DSC thermograms of the synthesized copolymers.

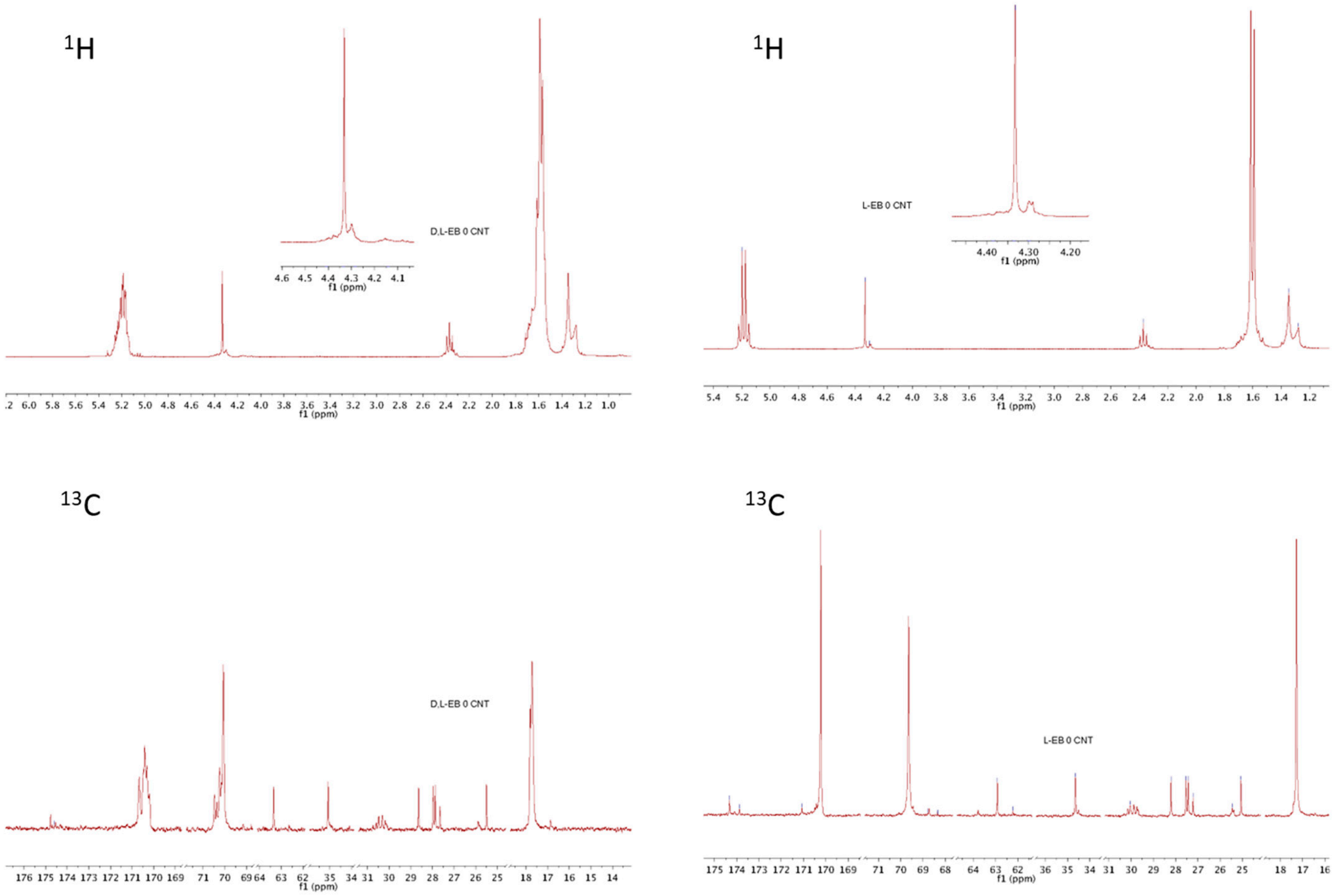

Figure S3. ^{1}H and ^{13}C NMR of synthetic (co)polymers.

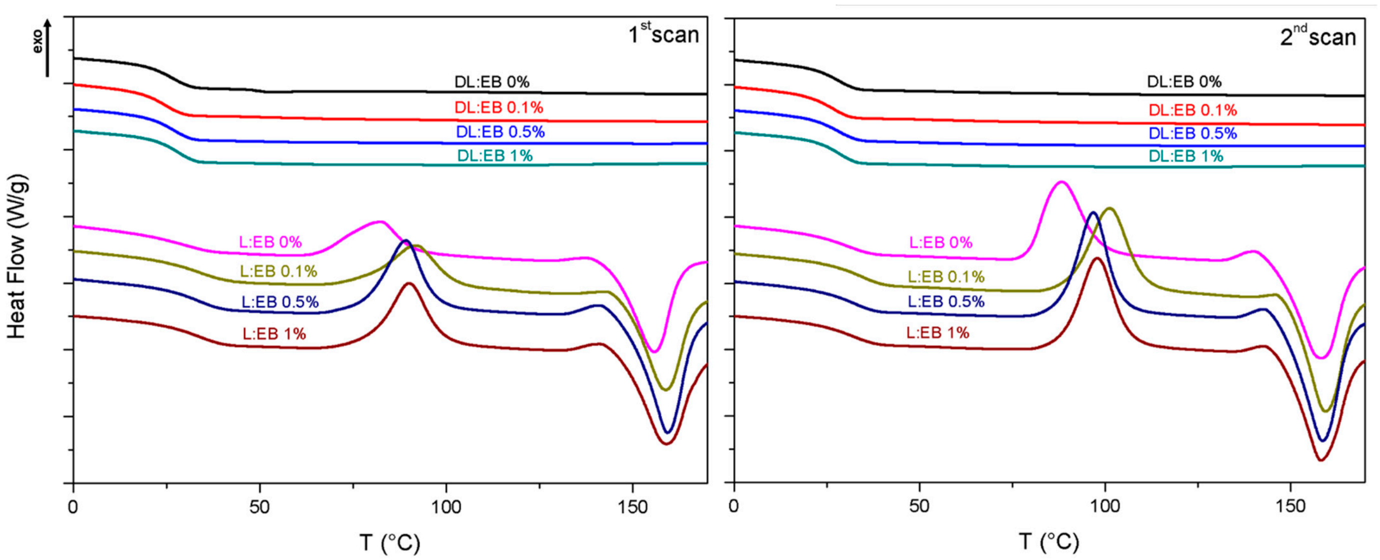

Figure S4. DSC thermograms of the final films.

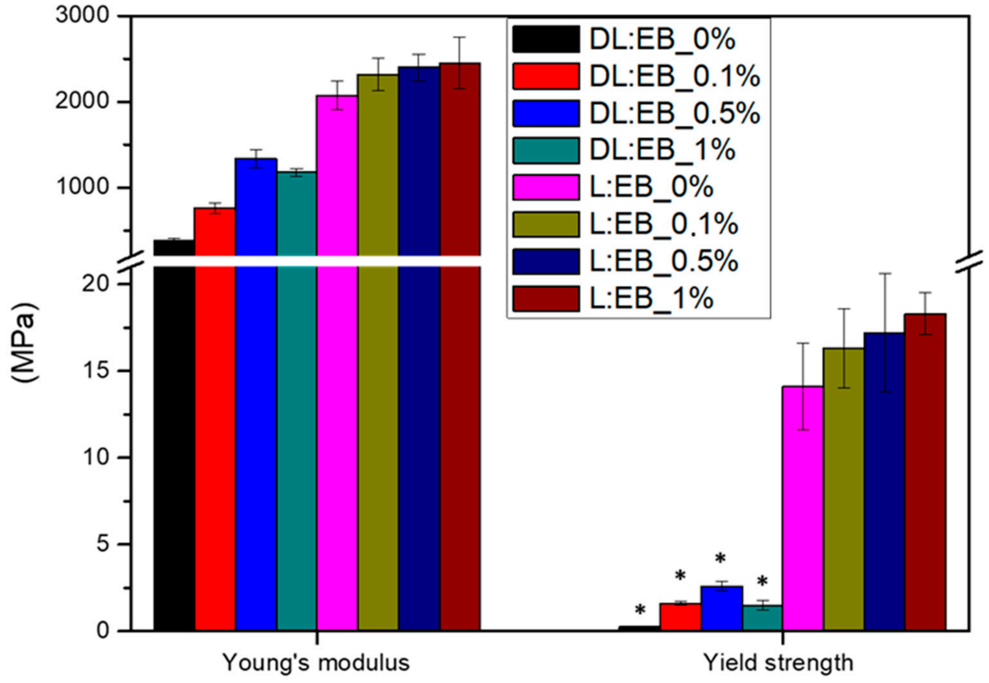

* Offset Yield strength was calculated at a 10% of strain using the secant modulus at 2% as elastic modulus (E)

Figure S5. Young's modulus (MPa) and Yield strength (MPa) of DL:EB and L:EB copolymers with different % of CNTs at ambient temperature (Ta).

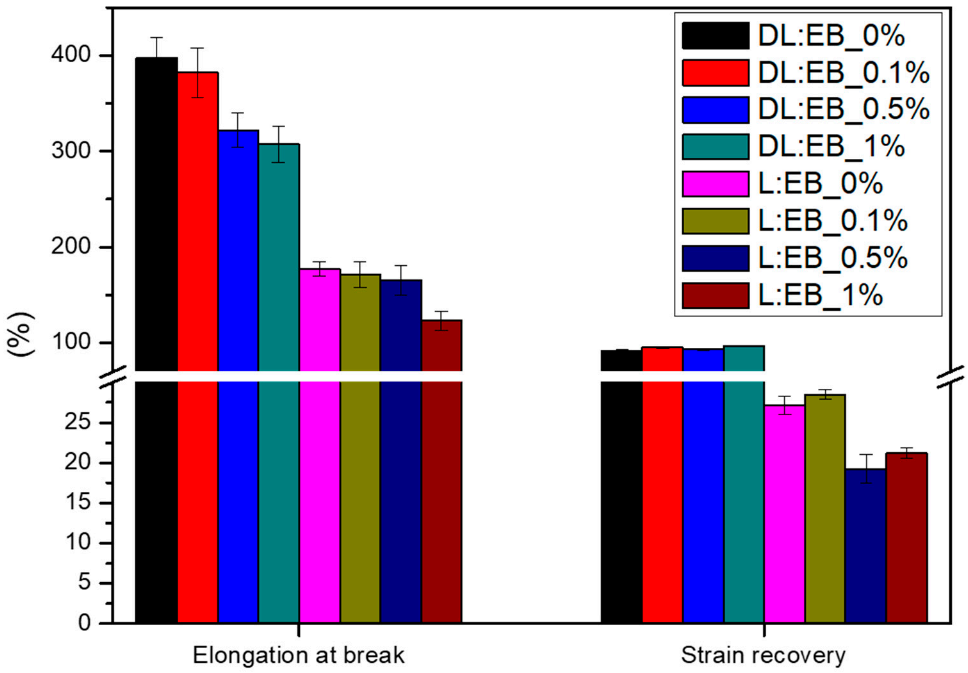

Figure S6. Elongation at break (%) and Strain recovery (%) of DL:EB and L:EB copolymers with different % of CNTs at ambient temperature (Ta).

Table S1. Crystallinity of L:EB films after 48 h at 37 °C submerged in culture media.

L:EB				
**%CNTs**	0%	0.1%	0.5%	1%
**χ (%) 0 h**	14.4	11.7	13.5	11.7
**χ (%) 48 h at 37°C**	30.4	31.1	31.3	31.7

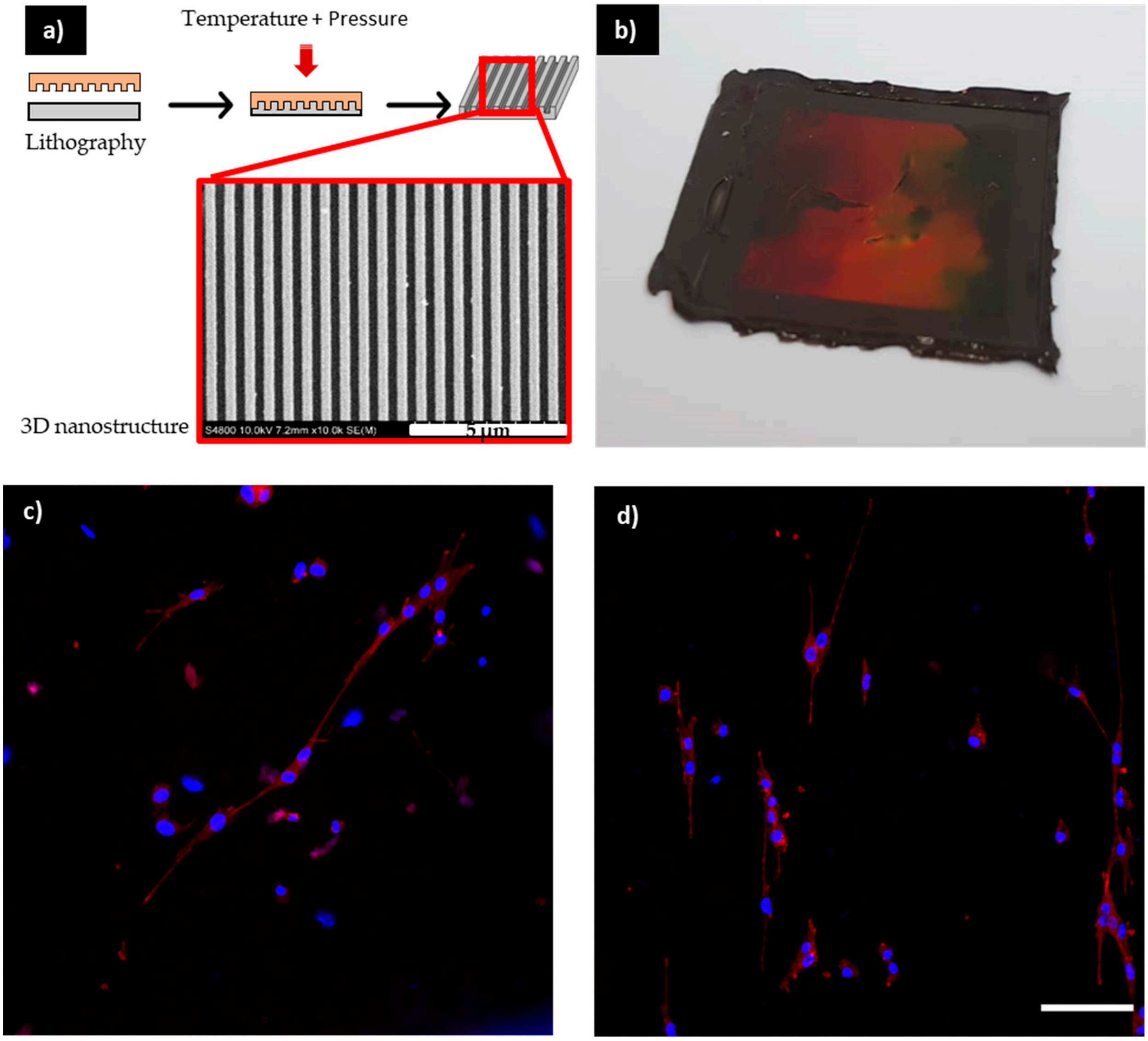

Figure S7. a) Schematic representation of the fabrication of the nanostructured film, b) SEM micrograph of the nanostructured film, c) C8-D1A seeded on nanostructured L:EB 0.5% CNTs film and d) C8-D1A seeded on nanostructured DL:EB 0.5% CNTs film. Scale bar 100 μm.
